# Bioinformatics Analysis Identifies Precision Treatment with Paclitaxel for Hepatocellular Carcinoma Patients Harboring Mutant *TP53* or Wild-Type *CTNNB1* Gene

**DOI:** 10.3390/jpm11111199

**Published:** 2021-11-13

**Authors:** Jiunn-Chang Lin, Tsang-Pai Liu, Vivin Andriani, Muhammad Athoillah, Chih-Yang Wang, Pei-Ming Yang

**Affiliations:** 1 Department of Surgery, MacKay Memorial Hospital, Taipei 10449, Taiwan; steven4375@gmail.com (J.-C.L.); liutp@mmh.org.tw (T.-P.L.); 2 MacKay Junior College of Medicine, Nursing, and Management, New Taipei City 11260, Taiwan; 3 Department of Medicine, MacKay Medical College, New Taipei City 25245, Taiwan; 4 Liver Medical Center, MacKay Memorial Hospital, Taipei 10449, Taiwan; 5 Ph.D. Program for Cancer Molecular Biology and Drug Discovery, College of Medical Science and Technology, Taipei Medical University and Academia Sinica, Taipei 11031, Taiwan; chihyang@tmu.edu.tw; 6 Department of Biological Science, Faculty of Science and Technology, Universitas PGRI Adi Buana, Surabaya 60234, Indonesia; v.andriani@unipasby.ac.id; 7 Department of Statistics, Faculty of Science and Technology, Universitas PGRI Adi Buana, Surabaya 60234, Indonesia; athoillah@unipasby.ac.id; 8 Graduate Institute of Cancer Biology and Drug Discovery, College of Medical Science and Technology, Taipei Medical University, Taipei 11031, Taiwan; 9 TMU Research Center of Cancer Translational Medicine, Taipei 11031, Taiwan; 10 Cancer Center, Wan Fang Hospital, Taipei Medical University, Taipei 11696, Taiwan

**Keywords:** bioinformatics, hepatocellular carcinoma, precision medicine, taxanes

## Abstract

Hepatocellular carcinoma (HCC) is an aggressive and chemoresistant cancer type. The development of novel therapeutic strategies is still urgently needed. Personalized or precision medicine is a new trend in cancer therapy, which treats cancer patients with specific genetic alterations. In this study, a gene signature was identified from the transcriptome of HCC patients, which was correlated with the patients’ poorer prognoses. This gene signature is functionally related to mitotic cell cycle regulation, and its higher or lower expression is linked to the mutation in tumor protein p53 (*TP53*) or catenin beta 1 (*CTNNB1*), respectively. Gene–drug association analysis indicated that the taxanes, such as the clinically approved anticancer drug paclitaxel, are potential drugs targeting this mitotic gene signature. Accordingly, HCC cell lines harboring mutant *TP53* or wild-type *CTNNB1* genes are more sensitive to paclitaxel treatment. Therefore, our results imply that HCC patients with mutant *TP53* or wild-type *CTNNB1* genes may benefit from the paclitaxel therapy.

## 1. Introduction

Primary liver cancer is still the sixth most common cancer type and the third leading cause of cancer-related death in the world [1]. Most (75–85%) of primary liver cancer cases involve hepatocellular carcinoma (HCC), whose major risk factors include chronic hepatitis B or C virus (HBV or HCV) infection, aflatoxin contamination in foods, excess body weight, heavy alcohol intake, smoking, and type 2 diabetes [1]. HCC development involves a complex, multi-step histological process of normal hepatocyte malignant transformation involving various genetic and epigenetic alterations [2]. The most frequent genetic alterations include mutations in telomerase reverse transcriptase (*TERT*) promoter, tumor protein p53 (*TP53*), and catenin beta 1 (*CTNNB1*) genes, as well as copy number variations and aberrations in DNA methylation [3]. *TERT* promoter mutations occur in dysplastic nodules and early HCC, and this gene is viewed as a gatekeeper for malignant transformation. *TP53* and *CTNNB1* mutations function as drivers during HCC development. Additional molecular alterations include focal DNA amplification (for example, vascular endothelial growth factor A/*VEGFA*, *MYC* proto-oncogene, bHLH transcription factor/*MYC*) and deletions (for example, cyclin-dependent kinase inhibitor 2A/*CDKN2A*, axin 1/*AXIN1*), and DNA methylation of promoter regions [3].

The current options for HCC management include surgical resection, liver transplantation, percutaneous local ablations (such as ethanol injection and radiofrequency thermal ablation), transarterial chemoembolization, transarterial radioembolization, and systemic pharmacological therapies [4,5]. The main curative treatments for HCC are surgical resection and liver transplantation, which are, however, only suitable for 15% to 25% of patients [6]. Furthermore, HCC is a chemoresistant and extremely refractory tumor type, and no reliable and effective treatments are available for those with advanced or metastatic disease [6]. Molecular targeted agents and immunotherapy have been regarded as treatment options in recent years. Although several multi-kinase inhibitors, such as sorafenib, regorafenib, lenvatinib, and cabozantinib, have been approved for treating advanced HCC [7,8,9,10], they only provide a short increase in median overall survival [7,8,10,11,12]. Immune checkpoint inhibitors, such as human anti-PD-1 monoclonal antibodies (nivolumab and pembrolizumab) and human anti-CTLA4 monoclonal antibody (ipilimumab), were approved for advanced HCC from 2017 to 2020, which greatly extend the patients’ overall survival [13,14,15].

Personalized or precision medicine has become a new trend in cancer treatment, which helps doctors select treatment for patients based on their genetic alterations [16]. Because HCC is a highly heterogeneous disease, grouping HCC patients into relatively homogeneous molecular subtypes may offer a significant clinical benefit through precision treatment [17]. HCC is usually classified based on tumor burden [18]. Recent advances in multi-omics technologies provide an opportunity for developing personalized treatment against HCC. For example, next-generation sequencing analyses of HCC identified new mutational signatures and defined new tumor subtypes that may benefit from targeted treatments in the future [19,20].

In this study, an integrated bioinformatics analysis determined that a prognostic mitotic gene signature is associated with *TP53*/*CTNNB1* mutation statuses in HCC. This gene signature could be targeted by paclitaxel, a clinically approved anticancer drug. Our results support that HCC patients with mutant *TP53* or wild-type *CTNNB1* genes may benefit from the paclitaxel anticancer therapy.

## 2. Materials and Methods

### 2.1. Cancer Genomics Analysis

Four microarray data sets from HCC patients, including GSE14520 [21], GSE45267 [22], GSE50579 [23], and GSE62232 [19], were obtained from the public Gene Expression Omnibus (GEO) depository database at the National Center for Biotechnology Information (NCBI). The differentially expressed genes (DEGs) between HCC tumor and adjacent normal tissues were obtained using the GEO2R online tool [24], and the criteria used to define DEGs were as follows: an adjusted *p*-value < 0.01 and |Log_2_ fold-change| > 1. The full DEG list is shown in File S1. The overlapped genes among four microarray data sets were visualized by a heat map generated using MORPHEUS software (https://software.broadinstitute.org/morpheus/; accessed on 21 January 2021). The prognostic values of these overlapped genes in HCC patients were further explored by GEPIA2 (http://gepia2.cancer-pku.cn/; accessed on 21 January 2021) [25] and/or cBioPortal (https://www.cbioportal.org/; accessed on 21 January 2021) [26,27], using The Cancer Genome Atlas (TCGA) hepatocellular liver carcinoma (LIHC) data set.

### 2.2. Pathway Enrichment Analysis

For pathway enrichment in Gene Ontology (GO) biological process, Kyoto Encyclopedia of Genes and Genomes (KEGG), and Reactome, selected genes were analyzed by STRING (https://string-db.org/; accessed on 21 January 2021), a database of known and predicted protein–protein interactions based on computational prediction, knowledge transfer between organisms, and interactions aggregated from other primary databases [28]. The following parameters were used: organism = *Homo sapiens*; network type = full network; network edges = evidence; active interaction sources = experiments and databases; minimum required interaction score = 0.4; max number of interactors to show = queried proteins only.

### 2.3. Gene–Drug Association Analysis

Gene–drug association was analyzed using GLAD4U (http://dlad4u.zhang-lab.org/; accessed on 26 January 2021) [29] via the WebGestalt website (http://www.webgestalt.org/; accessed on 26 January 2021) [30]. GLAD4U is a gene retrieval and prioritization tool based on existing biomedical literature [29]. WebGestalt is a functional enrichment analysis web tool that integrates various primary databases, including GLAD4U [30]. The gene–drug interaction network was further constructed using STITCH (http://stitch.embl.de/; accessed on 26 January 2021), a database of known and predicted interactions between chemicals and proteins based on computational prediction, knowledge transfer between organisms, and interactions aggregated from other primary databases [31]. The following parameters were used: organism = *Homo sapiens*; network edges = evidence; active interaction sources = experiments and databases; minimum required interaction score = 0.4; max number of interactors to show = queried proteins only.

### 2.4. Cancer Cell Drug Sensitivity Analysis

The drug sensitivity data, gene mutation status, and gene expression levels in HCC cell lines (Table S1) were downloaded from CellMinerCDB (https://discover.nci.nih.gov/cellminercdb/; accessed on 25 July 2021) [32] using the data in the Cancer Therapeutics Response Portal (CTRP; https://portals.broadinstitute.org/ctrp.v2.1/; accessed on 25 July 2021) [33,34,35]. The CTRP database links cancer cells’ genetic features to drug sensitivity [33,34,35], and CellMinerCDB is an interactive web-based tool allowing integration and analysis of genetic and pharmacological data in cancer cell lines across various data sets, including the CTRP [32].

## 3. Results

### 3.1. Identification of a Prognostic Mitotic Gene Signature in Hepatocellular Carcinoma

**Figure 1:**
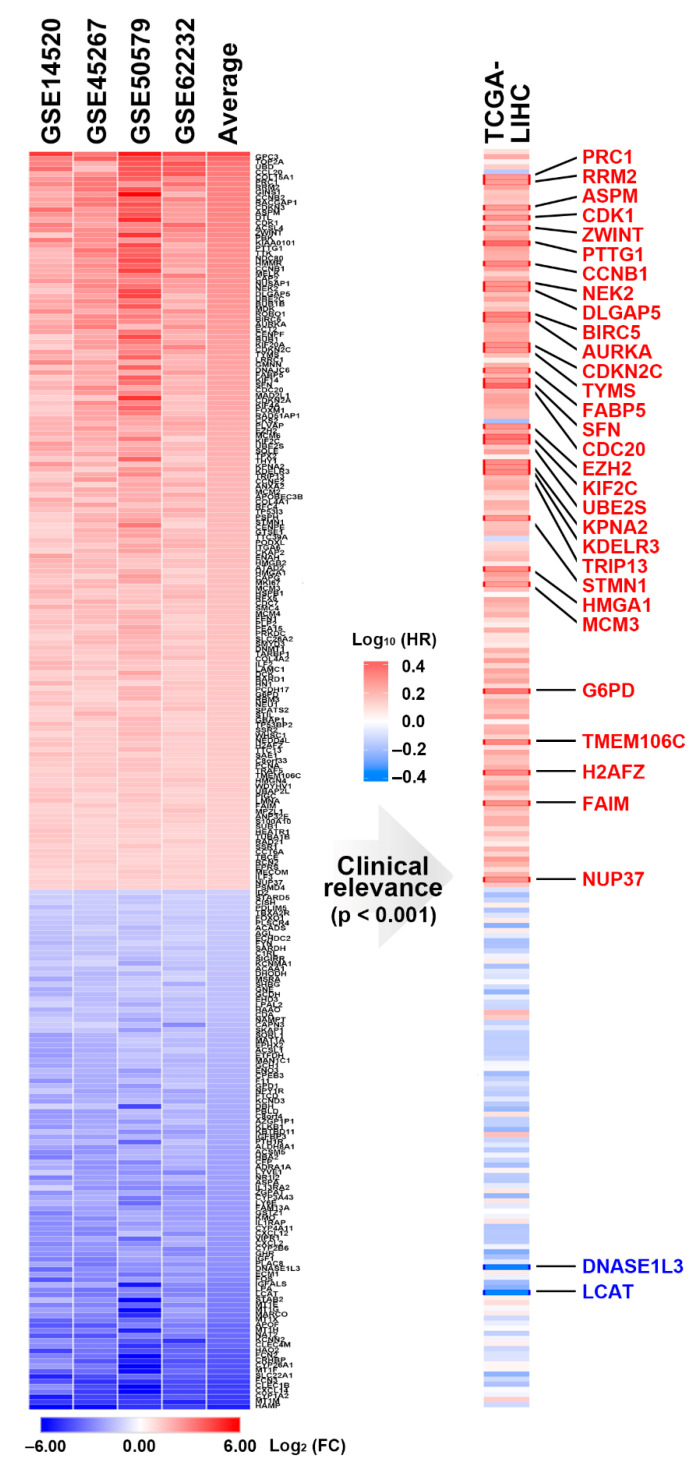
A prognostic gene signature for hepatocellular carcinoma (HCC). **Left**: The common gene signature in HCC was obtained from four data sets and ranked according to the average gene expression levels. **Right**: The prognostic impact of each gene in HCC was analyzed using the TCGA-LIHC data set. The genes highlighted in red or blue indicate these genes predicted better or poorer overall survival of HCC patients, respectively.

**Figure 2:**
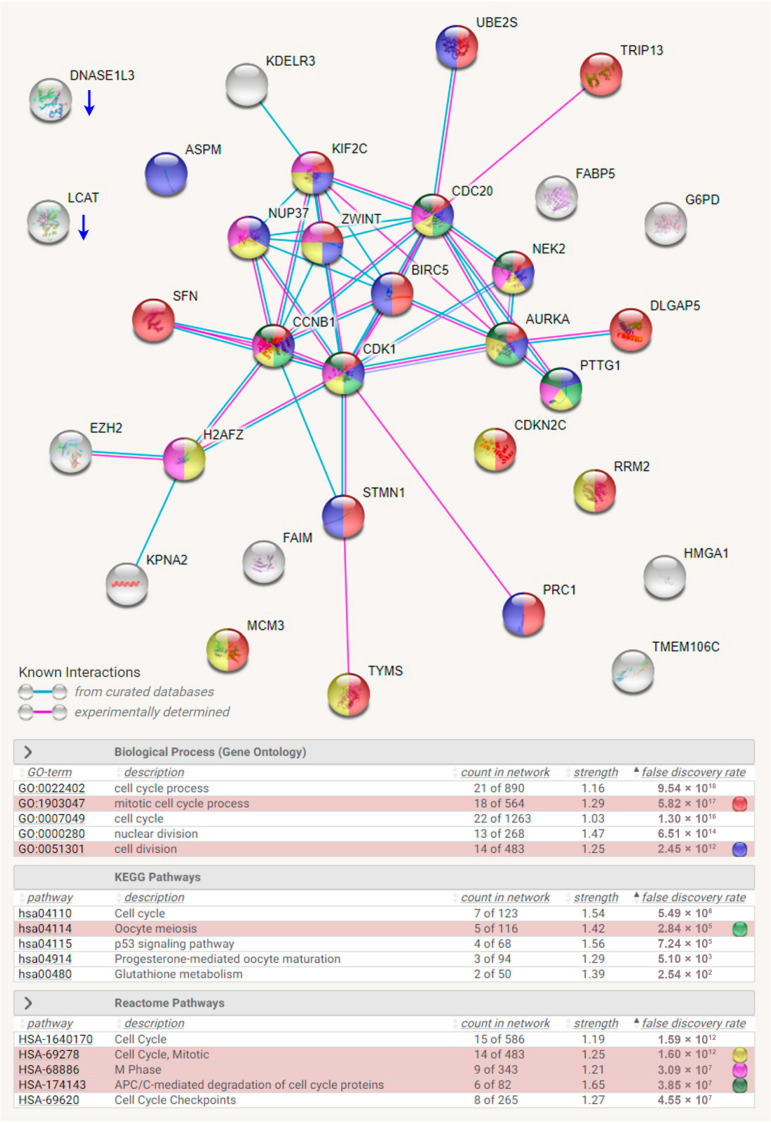
Pathway enrichment for the prognostic gene signature in hepatocellular carcinoma. The interaction network construction and pathway enrichment for the common gene signature in HCC was performed using the STRING database. The selected pathways are highlighted in different colors.

To identify a common gene signature in HCC patients, four microarray data sets (Table 1) were employed, and the common DEGs and their fold-change values are visualized in Figure 1 (left). To characterize the most essential genes in HCC patients, the prognostic roles of these DEGs in patients’ overall survival were analyzed using the TCGA-LIHC data set. As shown in Figure 1 (right), 30 upregulated and 2 downregulated genes were significantly linked to the patients’ overall survival. The interactions of these 32 genes were constructed by STRING [28]. Pathway enrichment results found that most of the upregulated genes are related to the cell cycle procession, especially mitosis (Figure 2). For example, mitosis is initiated by activating cyclin-dependent kinase 1 (CDK1) with its binding partner, cyclin B1 (CCNB1). Aurora kinase A (AURKA) and NIMA (never in mitosis gene A)-related kinase 2 (NEK2) are also mitotic kinases. Once activated, these mitotic kinases simultaneously or sequentially phosphorylate more than 1000 mitotic substrates (such as cell division cycle 20/CDC20 and pituitary tumor transforming gene 1/PTTG1) to regulate mitotic progression [36]. These results indicate that the commonly upregulated genes contributing to poor prognoses of HCC patients are correlated with the aberrant regulation of mitosis.

### 3.2. The Mitotic Gene Signature Is Associated with the Mutation Statuses of TP53 and CTNNB1 Genes

**Figure 3:**
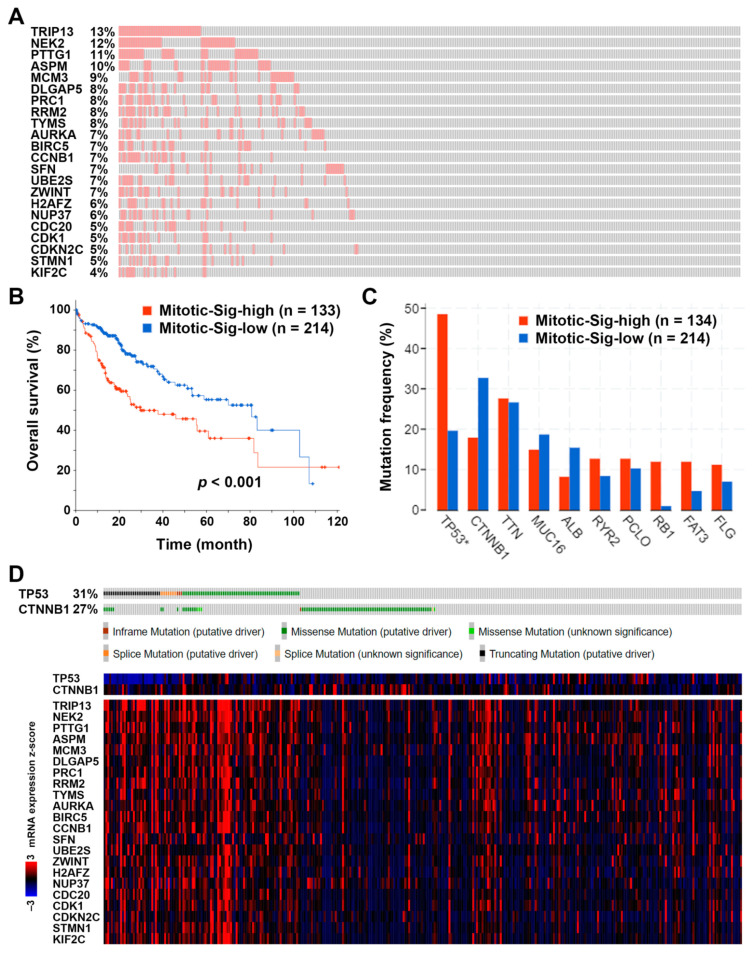
The association between the prognostic mitotic gene signature and gene mutations in hepatocellular carcinoma. (**A**) A waterfall plot shows the mRNA upregulation of the mitotic gene signature in HCC patients. (**B**) The prognostic impact of the mitotic gene signature expression on the overall survival of HCC patients. (**C**) The gene mutation frequency in HCC patients with high or low expression levels of mitotic gene signature. * indicates significant differences (*p* < 0.05) between two groups. (**D**) Upper: The genetic mutation statuses of *TP53* and *CTNNB1* genes in HCC patients. (**D**) Lower: The heat map for the mRNA expression levels of *TP53*, *CTNNB1*, and mitotic gene signature.

The 22 genes (mitotic gene signature) annotated to the cell cycle and mitosis (Figure 2) were further analyzed using the TCGA-LIHC data for their mRNA expression levels. Among 348 HCC patients, 134 (38.5%) exhibited higher expression of mitotic gene signature (Figure 3A). Consistent with the result in Figure 1 (right), patients with higher mitotic gene signature expression had poorer overall survival (Figure 3B). Therefore, this mitotic gene signature may be utilized for the prediction of HCC patients’ prognoses.

Interestingly, the higher mitotic gene signature expression significantly correlated with *TP53* gene mutation (Figure 3C). According to the TCGA-LIHC data set, 31% of HCC patients harbored *TP53* mutations (including missense, truncating, splicing, and inframe mutations), and they tended to have higher mitotic gene signature expression (Figure 3D). We also noticed that HCC patients harboring *CTNNB1* mutations seemed to have lower mRNA expression levels of the mitotic gene signature, although no statistically significant differences existed (Figure 3C,D). Given the finding that *TP53* and *CTNNB1* gene mutations usually occur in a mutually exclusive manner in HCC [3], the overexpression of this mitotic gene signature may result from the *TP53* mutation during HCC development.

### 3.3. Paclitaxel Provides Therapeutic Benefit for Hepatocellular Carcinoma Cells Harboring Mutant TP53 or Wild-Type CTNNB1 Genes

**Figure 4:**
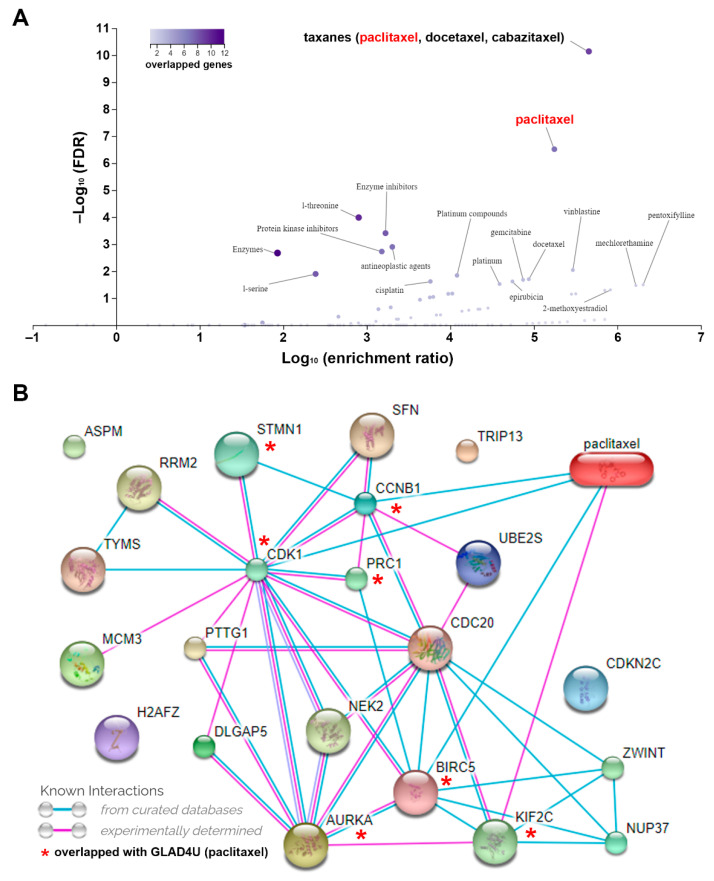
Prediction of potential drugs targeting the mitotic gene signature. (**A**) The association between drugs and mitotic gene signature. (**B**) The network between paclitaxel and mitotic gene signature.

To identify potential drugs that targeted the mitotic gene signature, we used the GLAD4U web-based tool to search drug-gene interactions [29]. As shown in Figure 4A, taxanes (paclitaxel, docetaxel, and cabazitaxel) were the most significant drugs linking to the mitotic gene signature. As a representative, the paclitaxel-gene interacting network was further constructed using the STITCH database [31]. The common genes associated with paclitaxel were CDK1, CCNB1, baculoviral IAP repeat containing 5 (BIRC5), kinesin family member 2C (KIF2C), stathmin 1 (STMN1), protein regulator of cytokinesis 1 (PRC1), and AURKA (Figure 4B). Taxanes belong to microtubule-stabilizing agents that cause inhibition of mitosis by preventing the degradation of microtubules [37]. Thus, it is reasonable to say that paclitaxel could target aberrant mitosis in HCC.

**Figure 5:**
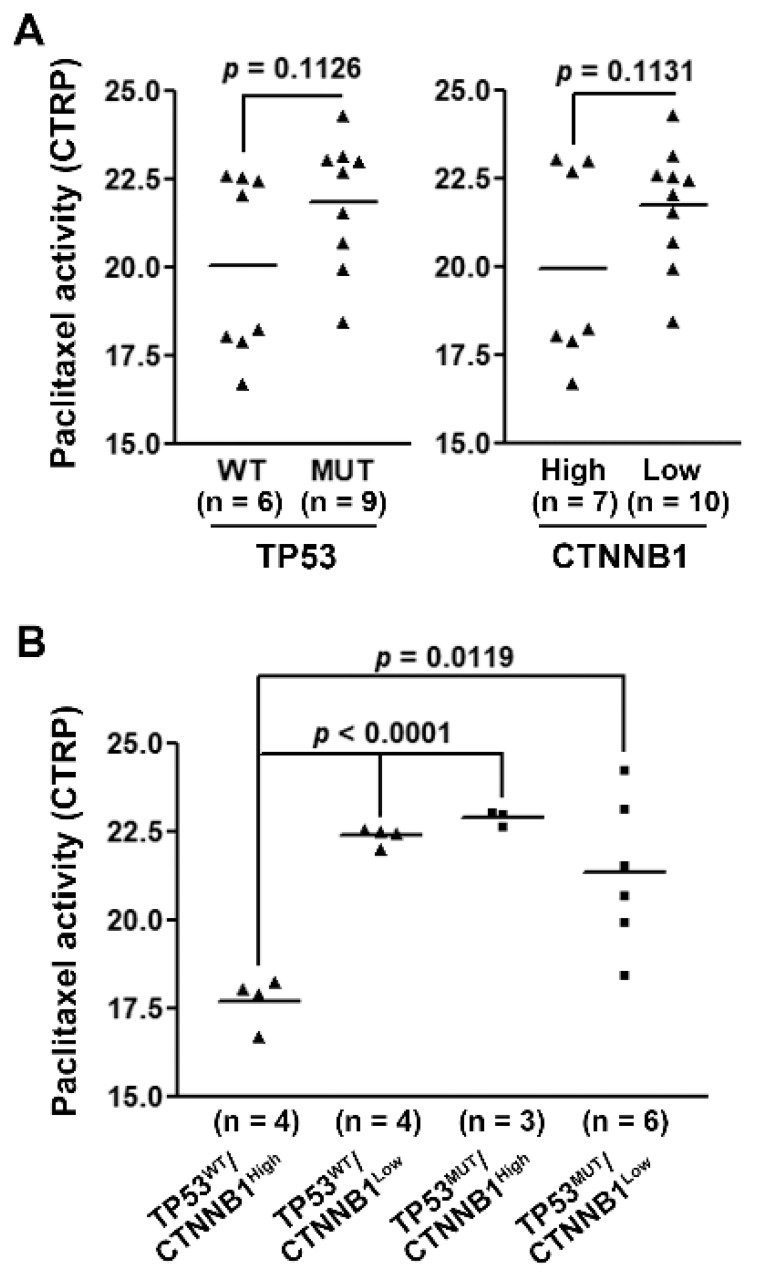
The role of *TP53* and *CTNNB1* gene statuses in the paclitaxel drug sensitivity in hepatocellular carcinoma cells. The paclitaxel drug activity in HCC cell lines according to the *TP53* mutation status (**A**, left), *CTNNB1* mRNA expression (**A**, right), or their combination (**B**).

Because the mitotic gene signature correlated with *TP53* and *CTNNB1* gene mutations in HCC patients (Figure 3C,D), we hypothesized that HCC subtyping based on *TP53* and *CTNNB1* gene mutation statuses might exhibit a potential benefit from paclitaxel treatment. To demonstrate this possibility, the relationship between drug activity and gene mutations in HCC cell lines was retrieved from the CTRP database [33,34,35]. However, only one HCC cell line harbored *CTNNB1* gene mutation (Figure S1A). Because *CTNNB1* mutations were associated with higher *CTNNB1* mRNA expression levels in HCC patients (Figure S1B), we used *CTNNB1* gene expression levels to compare with paclitaxel drug activity in HCC cell lines. As shown in Table S1, HCC cells divided by either *TP53* mutation status or *CTNNB1* expression level exhibited no significantly different sensitivity to paclitaxel treatment (Figure 5A). Interestingly, HCC cells simultaneously harboring wild-type *TP53* and higher *CTNNB1* expression were the most resistant to paclitaxel (Figure 5B), confirming that higher mitotic gene signature expression contributes to the increased sensitivity to paclitaxel.

## 4. Discussion

Because HCC is highly aggressive and resistant to chemotherapy, novel therapeutic strategies are still urgently needed. Precision medicine is a new and emerging strategy that helps doctors better understand disease and provide new options for patients. However, as novel medicines will not benefit all patients, finding biomarkers is critical. Cancer bioinformatics is one of the useful tools aiding in the discovery of biomarkers and the prediction of therapy efficacy. In this paper, we present a successful example of using bioinformatics tools to uncover biomarkers for the precision HCC treatment.

According to our findings, the overexpression of mitotic gene signature is associated with poorer overall survival and *TP53* gene mutation in HCC patients. The overexpression of mitotic genes may represent abnormal mitosis that is required for rapid tumor growth. Consistently, a higher mitotic index is observed in HCC patients and is associated with their poorer prognoses [38,39,40]. In addition, aberrant p53 protein expression is associated with higher mitotic and proliferative indexes in HCC tumors [41], further supporting our results. However, our conclusion may be limited by the HCC patients’ clinical statuses. For example, a localized or advanced stage may also contribute to HCC patients’ prognoses, which should be further clarified in-depth.

Immunotherapy, such as immune checkpoint inhibitors, has become a promising new strategy for treating advanced HCC [42]. Between 2017 and 2020, two human anti-PD-1 monoclonal antibodies (nivolumab and pembrolizumab) and an anti-CTLA4 monoclonal antibody (ipilimumab) were approved for advanced HCC, extending patients’ overall survival significantly [13,14,15]. Higher tumor mutation burden (TMB-H) has been viewed as a predictive biomarker for better responses of cancer patients to anti-PD-1 and anti-CTLA4 therapy, because the increasing numbers of mutant-protein-derived tumor antigens allow the enhanced immunogenicity [43]. The *TP53* gene is one of the most frequent driver mutations during HCC development [3] and is positively correlated with TMB-H [44,45]. Because the mitotic gene signature is positively associated with *TP53* gene mutation in HCC, this signature may be used to predict the efficacy of immunotherapy. Supportively, a higher immune cell infiltration is associated with a higher mitotic index in gastrointestinal stromal tumors [46].

Mutations in *TP53* and *CTNNB1* genes in HCC usually occur in a mutually exclusive manner [3]. In contrast to *TP53* mutation, *CTNNB1* mutation has been found to define immune-cold HCC, meaning that *CTNNB1*-mutated HCC tends to be refractory to immune checkpoint inhibitors [47,48]. Our results also showed that the overexpression of mitotic gene signature is negatively associated with *CTNNB1* gene mutation, which further supports the predictive value of the mitotic gene signature to immune checkpoint inhibitors.

Taxanes are widely used chemotherapy for many cancers [49,50]. For example, the nanoparticle albumin-bound-paclitaxel is approved for treating breast cancer, non-small cell lung cancer, and pancreatic cancer. Docetaxel is approved for treating breast cancer, non-small cell lung cancer, and prostate cancer. Cabazitaxel is approved for treating prostate cancer. However, taxanes have not yet been approved for HCC. Only a few clinical studies for taxanes in HCC have been previously performed. Phase II trials of paclitaxel and docetaxel have not produced satisfactory results [51,52] in unresectable and advanced HCC patients, respectively. Our bioinformatics analysis may provide a basis for further clinical trial designs of taxanes for HCC using *TP53*/*CTNNB1* mutation as biomarkers. However, one limitation is that only a few HCC cell lines with different *TP53*/*CTNNB1* gene statuses were used to predict sensitivities to paclitaxel treatment. More HCC cell lines, or more clinically relevant patient-derived organoids, should be used to confirm these results.

The major effect of taxanes is to stabilize microtubules [37]. Our analysis showed that the main targets for paclitaxel are CDK1, CCNB1, BIRC5, KIF2C, STMN1, PRC1, and AURKA. Several of these are associated with microtubules. For example, STMN1 is a microtubule-destabilizing protein whose expression is upregulated by gain-of-function mutant p53 during HCC development. Loss of STMN1 sensitizes HCC tumor cells to microtubule-targeting agents, including paclitaxel [53]. PRC1 is a microtubule-binding protein regulating cytokinesis. Inhibition of PRC1 and paclitaxel treatment exhibits synergistic anticancer activity against HCC [54]. KIF2C, a mitotic centromere-associated kinesin, is a microtubule-based motor protein that serves as a therapeutic target for HCC [54]. BIRC5, also known as survivin, is a microtubule-associated protein and participates in the assembly of the bipolar mitotic spindle [55]. Therefore, targeting the mitotic gene signature in HCC may influence microtubule dynamics, leading to the enhancement in taxane’s anticancer activity.

Drug discovery is still a risky, time-consuming, and expensive process, with only a few novel anticancer medications receiving approval in recent years [56,57,58]. Drug repurposing is an unconventional strategy that uses existing therapeutic drugs to treat new disease indications or specific patient populations [59,60,61]. Drug repurposing has several advantages, including cost savings and bypassing safety concerns, since extensive drug data are frequently available [59,60,61]. Because taxanes have been used as anticancer chemotherapy for decades [49,50], repurposing taxanes such as paclitaxel to treat HCC would be a feasible strategy.

## 5. Conclusions

In conclusion, we used bioinformatic approaches to establish a predictive mitotic gene signature in HCC linked to the mutation statuses of the *TP53* and *CTNNB1* genes. We also discovered that paclitaxel, a clinically approved anticancer drug, can be utilized to treat HCC patients with mutant *TP53* or wild-type *CTNNB1* genes, providing a precision treatment option for the future.

## Figures and Tables

**Table 1:** Microarray data sets for hepatocellular carcinoma patients.

Access Number	Platform	Normal	Tumor	References
GSE14520	Affymetrix Human Genome U133A 2.0	21	22	[21]
GSE45267	Affymetrix Human Genome U133 Plus 2.0	39	48	[22]
GSE50579	Agilent SurePrint G3 Human GE 8 × 60 K	10	67	[23]
GSE62232	Affymetrix Human Genome U133 Plus 2.0	10	81	[19]

## Data Availability

The data supporting this study can be obtained from the public databases or are available on request from the corresponding author.

## Supplementary Materials

The following are available online at https://www.mdpi.com/article/10.3390/jpm11111199/s1, Figure S1: The correlation between *CTNNB1* gene mutation and mRNA expression levels in CTRP-HCC (A) and TCGA-LIHC (B) data sets, Table S1: The *TP53* and *CTNNB1* gene statuses in HCC cell lines, File S1: The differentially expressed genes (DEGs) prepared from microarray data sets.
