# Supplementary material for: Bioinformatics Analysis Identifies Precision Treatment with Paclitaxel for Hepatocellular Carcinoma Patients Harboring Mutant TP53 or Wild-Type CTNNB1 Gene
Source: J Pers Med. 2021 Nov 13;11(11):1199. doi: 10.3390/jpm11111199 (PMC8623741; doi:10.3390/jpm11111199)
Supplement: Supplementary file 1 [file jpm-11-01199-s001.zip › JPM-Supplementary_Material.pdf]

Supplementary Material

Table S1. The TP53 and CTNNB1 gene statuses in HCC cell lines.

Cell Line	TP53 mutation	CTNNB1 mutation	CTNNB1 expression	CTNNB1 classification	Paclitaxel activity
HepG2	WT	WT	7.602243362	High	17.892
JHH-6	WT	WT	8.63738242	High	16.687
SK-HEP-1	WT	WT	7.970566444	High	18.259
SNU-761	WT	WT	8.138213995	High	18.041
SNU-182	MUT	WT	7.65798652	High	22.6784
SNU-878	MUT	WT	8.329187639	High	23.0402
SNU-886	MUT	WT	8.292320426	High	22.9885
JHH-1	WT	WT	6.830246622	Low	22.0308
JHH-2	WT	WT	7.428550153	Low	22.5771
JHH-5	WT	WT	7.341289808	Low	22.5304
Li-7	WT	WT	7.240726404	Low	22.4692
HLF	MUT	WT	7.165552354	Low	23.1611
JHH-4	MUT	WT	7.565088591	Low	21.5418
SNU-387	MUT	WT	7.102336472	Low	20.7236
SNU-423	MUT	WT	7.424584171	Low	24.2819
SNU-449	MUT	WT	6.942420508	Low	18.442
SNU-475	MUT	WT	7.238673665	Low	19.9617

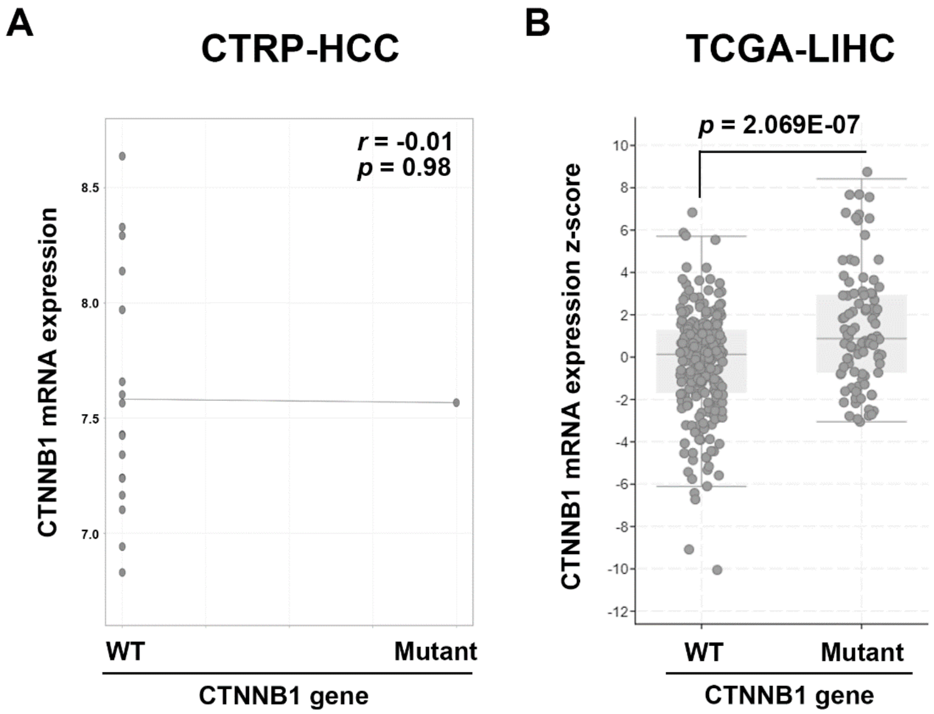

Figure S1. The correlation between *CTNNB1* gene mutation and mRNA expression levels in CTRP-HCC (A) and TCGA-LIHC (B) data sets.
